# Prior Cereal Leaf Beetle (*Oulema* spp.) Window-Pane Feeding Has Weak Systemic Legacy on the Wheat Dwarf Virus Vector *Psammotettix alienus* in Barley

**DOI:** 10.3390/insects17080783

**Published:** 2026-07-28

**Authors:** Regina Gerstenbrand, Zoltán Tóth, Ferenc Samu, Gergely Tholt

**Affiliations:** HUN-REN Centre for Agricultural Research, Plant Protection Institute, 1518 Budapest, Hungary; gerstenbrand.regina@atk.hun-ren.hu (R.G.); toth.zoltan@atk.hun-ren.hu (Z.T.)

**Keywords:** plant-mediated interactions, barley, sequential herbivory, electrical penetration graph, EPG, phloem feeding, vector behaviour

## Abstract

Plant-mediated interactions can modify herbivore behaviour and vector-borne plant disease dynamics, yet the systemic consequences of prior cereal leaf beetle (*Oulema* spp.) window-pane feeding for later phloem feeder vectors remain unclear. We hypothesised that prior *Oulema* window-pane feeding induces systemic changes in barley that alter the subsequent host acceptance and feeding behaviour of *Psammotettix alienus*. Plants received one of three 24 h treatments on one leaf: *Oulema* pre-colonisation, standardised artificial vascular injury or an undamaged control. Leafhopper responses were measured on a different, systemic leaf using female choice assays and electrical penetration graph recordings. *Oulema* damage was low and strongly right-skewed and did not affect most feeding variables, including non-phloem activities, phloem activity, drinking latency or phloem activity latency. Artificial vascular injury, however, reduced the phloem–saliva ratio in females, which indicates a lower relative investment in phloem preparation once the phloem phase was reached. Within *Oulema*-treated plants, increasing damage reduced the number of transitions between penetration phases in males but not females. Females showed no treatment-related preference in salivary sheath production or oviposition. Thus, *Oulema* window-pane feeding left only a weak systemic plant-mediated legacy for P. alienus, closer to behavioural neutrality than strong facilitation or interference under these short-term systemic experimental conditions.

## Simple Summary

In cereal fields, different insect pests often feed on the same plants in sequence, and damage by one species may change how later pests use the plant. We tested whether previous feeding by cereal leaf beetles changes the behaviour of *Psammotettix alienus*, a leafhopper that transmits wheat dwarf virus. Barley plants were first exposed for 24 h to cereal leaf beetle feeding, to a controlled cut that damaged leaf veins, or to no damage. We then measured whether female leafhoppers preferred leaves from differently treated plants and how leafhoppers fed on untreated leaves of the same plants. Previous cereal leaf beetle feeding caused little change in leafhopper behaviour. Females did not prefer treated or untreated plants for feeding or egg laying, and most feeding variables were unchanged. A controlled vein injury caused a small shift in the balance of phloem-related feeding activities, while greater beetle damage slightly reduced switching among feeding phases in males. Overall, short-term cereal leaf beetle feeding left only a weak systemic effect on this virus vector, which suggests that moderate window-pane feeding is unlikely to strongly alter wheat dwarf virus risk through leafhopper feeding behaviour alone.

## 1. Introduction

Phytophagous insect communities have traditionally been interpreted through the lens of interspecific competition, under the assumption that herbivores sharing a host plant compete for limited nutritional resources. However, classic competition theory often fails to predict the outcome of insect herbivore interactions, largely because insect–plant interactions are highly asymmetrical and strongly mediated by host plant responses [1,2]. Insect feeding can alter plant nutritional quality, resource allocation and defence expression [3]. These induced responses often extend beyond the initial plant–herbivore interaction, shaping subsequent interactions with other herbivores, natural enemies, and plant pathogens—a phenomenon collectively termed “plant-mediated interactions” [2,4]. Such plant-mediated interactions, even in the absence of physical contact, may result in competition, facilitation or near-neutrality depending on herbivore identity, feeding guild, feeding intensity and the sequence of arrival [4,5]. However, a key unresolved question is how these plant-mediated effects cascade from individual feeding events to broader ecological dynamics.

Plant-mediated effects are often highly dependent on the feeding strategy and mouthpart morphology of the attacking insects, because chewing and piercing–sucking insects differ in both the type of damage they impose and the defensive pathways they typically activate [6]. Chewing insects directly remove photosynthetically active plant tissue, whereas sap-sucking insects generally leave this tissue structurally intact. Salivary sheath feeding is a form of piercing–sucking herbivory in which stylet movement, salivation and sap uptake are functionally distinct. During penetration, gelling saliva forms a sheath around the stylets, while watery saliva is secreted during pathway activities and into sieve elements before sustained phloem ingestion. This phloem salivation can suppress wound responses, thereby facilitating sustained sap uptake [7,8,9]. These feeding phases differ in their costs and nutritional benefits: pathway activities, sheath formation and salivation require time and physiological investment but provide little direct nutrition. Xylem ingestion supports water balance under the osmotic stress imposed by sugar-rich phloem sap, whereas sustained phloem ingestion is the main nutrient-acquisition phase, providing carbohydrates and nitrogen-containing compounds [10]. Thus, the preparatory phases represent the principal behavioural and physiological investment preceding nutrient uptake.

The plant-mediated effects of multiple herbivory are strongly shaped by the colonisation sequence [5,11,12]. Early arriving herbivores, pre-colonisers, can impose priority effects by redirecting the host’s metabolic trajectory, thereby altering plant quality, defence expression and resource availability for later colonisers [2,4]. Prior chewing herbivory can either increase or reduce plant resistance to later phloem feeders, depending on the defence response involved [13,14,15]. Thus, the consequences of prior herbivory for later sap feeders are best viewed as context-dependent plant-mediated effects rather than predictable outcomes of feeding guild alone; they also depend on the arrival sequence, the tissue damaged and the defence mechanisms induced.

Agroecosystems are frequently colonised in succession by pest species from different feeding guilds, which makes cereals suitable systems for studying such interactions. Among the most widespread and economically significant pests in temperate cereal production are cereal leaf beetles (CLBs) of the genus *Oulema* (Coleoptera: Chrysomelidae) and the leafhopper, *Psammotettix alienus* (Hemiptera: Cicadellidae). In European agroecosystems, these two taxa frequently co-occur and share host plants such as barley (*Hordeum vulgare*) and wheat (*Triticum aestivum*) [16]. *Oulema* species cause substantial direct damage through their characteristic “window-pane feeding”, which removes mesophyll and epidermal tissue between leaf veins, reducing the photosynthetically active leaf area and depressing yield [17,18]. This feeding mode is not equivalent to generic chewing damage, which often results in vascular tissue damage or complete defoliation. Nevertheless, *Oulema* feeding can induce diverse physiological and chemical defence responses [19,20,21]. Moreover, authentic *Oulema* feeding is not equivalent to sterile mechanical injury either: it can induce broader volatile responses than mechanical damage simulating the window-pane effect, while herbivore-associated microbiota may further modulate wheat defence expression [22,23]. Thus, despite causing less direct vascular disturbance than vein-breaching injury, *Oulema* feeding may still leave a complex plant-mediated legacy for later sap feeders.

The other key herbivore in this system, *P. alienus*, is an oligophagous salivary sheath leafhopper primarily associated with Poaceae. Its direct feeding damage is relatively minor, but its epidemiological importance is high because it is the principal vector of wheat dwarf virus (WDV), a phloem-restricted mastrevirus that is persistently transmitted and can cause severe yield losses in cereals [24,25]. Previous studies show that *P. alienus* participates in plant- and insect-mediated interactions within cereal sap-feeder communities: *Rhopalosiphum padi* can negatively affect its development and reproduction, whereas previous *P. alienus* infestation can decrease the fitness of *Sitobion avenae* [26,27,28]. However, these studies do not address whether prior window-pane feeding by a leaf herbivore alters the subsequent feeding behaviour of the leafhopper. Because virus inoculation is associated with phloem salivation, whereas virus acquisition is associated with phloem ingestion [25], changes in the allocation of feeding time between these phases may have epidemiological consequences.

A major challenge in studying the piercing–sucking feeding process is that within-plant activities cannot be directly observed. The electrical penetration graph (EPG) technique overcomes this limitation by recording in vivo waveform patterns associated with different phases of the feeding process [29,30,31]. Recorded waveforms can be grouped into biologically meaningful variables representing different phases of the feeding process, including activities involved in counteracting plant defences, fulfilling physiological needs such as drinking, acquiring nutrients and mediating pathogen transmission. Previous EPG studies have shown that *P. alienus* adjusts different phases of its feeding behaviour in response to host suitability, as shown by comparisons between host and non-host plants, and ecological factors such as predator-related stress [25,30]. Whether the plant-mediated legacy caused by prior *Oulema* feeding is sufficient to shift the same functional feeding components remains unknown.

Despite their frequent coexistence, the plant-mediated interactions between the contrasting feeding strategies of *Oulema* spp. and *Psammotettix alienus* remain poorly understood. In particular, the indirect effects of *Oulema* window-pane feeding—a widespread form of herbivory distinct from vein-breaching chewing damage—on the subsequent host use of a sap-feeding leafhopper remain unknown [18]. Therefore, the main question of our study is whether prior *Oulema* spp. damage results in behavioural evidence consistent with interference, facilitation or behavioural neutrality for the subsequent coloniser. We examined the systemic effects of three treatments: (a) prior *Oulema* window-pane feeding; (b) a standardised vascular injury treatment designed to contrast vein-breaching damage with interveinal window-pane feeding; and (c) an undamaged control. Using choice assays and electrical penetration graph recordings, we asked whether these treatments alter host plant choice for feeding and oviposition, phloem access and the allocation of feeding time among phloem-associated phases, some of which are also relevant to virus transmission. We interpreted delayed phloem access, increased salivation investment or reduced phloem activity as evidence consistent with feeding interference through increased systemic resistance, whereas faster access, reduced salivation investment or increased host acceptance for feeding and oviposition would indicate facilitation.

## 2. Materials and Methods

### 2.1. Plants and Insects

Barley plants (*Hordeum vulgare* cv. Mv. Conchita) and the stock population of *Psammotettix alienus* were maintained at 21 °C under an L16:D8 photoperiod. Adult cereal leaf beetles were collected from an experimental barley plot and acclimated under the same conditions before the assays. To provide an indication of the species composition of the *Oulema* population, 135 randomly selected specimens were identified: 83% were *O. melanopus*, 11% were *O. duftschmidi*, and 6% were *O. rufocyanea*. Detailed plant and insect maintenance procedures, including leafhopper stock rearing and beetle collection and acclimation, are provided in Supplementary Materials S1.

### 2.2. Treatments

*Oulema* pre-colonisation treatment procedures were implemented identically across the choice and feeding behaviour assays. Experimental plants were grown individually in 200 mL pots until the three-leaf stage, and treatments were applied consistently to the second leaf. To standardise the treated area and prevent insect escape, a custom micro-isolator was attached to the second leaf of each plant. Details of micro-isolator construction and use are provided in Supplementary Materials S2.

**Figure 1:**
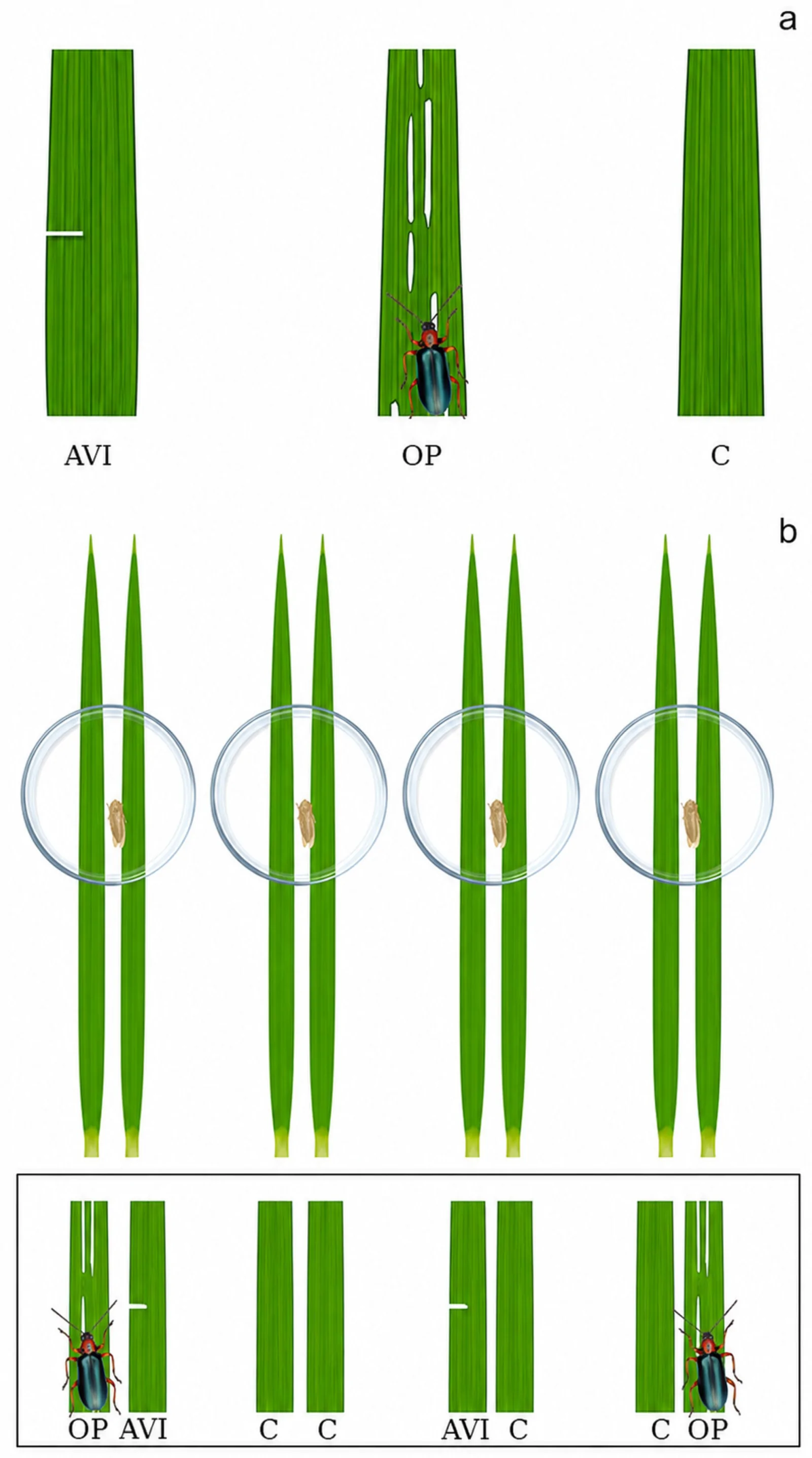
Overview of the pre-colonisation treatments used across all tests and the experimental set-up of the female choice experiment assessing leafhopper feeding and egg-laying preferences. (**a**) Schematic overview of the treatments: AVI involved a standardised transverse vein-breaching cut, OP involved *Oulema* feeding and controls were untreated. (**b**) Experimental set-up of the female choice experiment. Female leafhoppers were individually confined for 24 h in a circular choice arena on paired systemic leaves from differently treated plants to assess feeding and egg-laying preferences across C–OP, C–AVI, C–C and AVI–OP treatment combinations.

We applied three treatments: *Oulema* pre-colonisation (OP), artificial vascular injury (AVI) and an undisturbed control (C) (Figure 1). As a procedural control, micro-isolators were attached in all treatments, irrespective of beetle presence. To minimise cross-treatment exposure to induced volatiles before the assays, plants assigned to different treatments were kept in separate rooms until trials began [22]. In the OP treatment, one adult cereal leaf beetle was introduced into the micro-isolator and allowed to feed on the enclosed leaf section. The AVI treatment was designed as a standardised vein-breaching injury rather than as a mechanical surrogate for authentic *Oulema* feeding. A single transverse cut was made with sharp scissors perpendicular to the midrib, severing the smaller parallel veins on one side of the leaf while leaving the midrib intact.

Treatments lasted for 24 h, with micro-isolators remaining in place for the full duration in all groups. All assays were initiated immediately after this treatment period. Recordings and observations were conducted on the untreated systemic third leaf rather than on the treated second leaf, allowing us to evaluate systemic plant-mediated effects [2]. Across all treatments and assays, test animals were randomly selected from the source population and used only once.

Following the completion of the choice and feeding behaviour tests, the pre-colonised leaves from the beetle treatment (OP) were photographed to quantify the extent of *Oulema* damage. *Oulema* damage was quantified from digital images using ImageJ v.1.53 (National Institutes of Health, Bethesda, MA, USA) [32] and expressed as the percentage of damaged leaf area; this value was used as the ‘damage intensity’ variable. Details of image acquisition, back-illumination and damage delineation are provided in Supplementary Materials S4.

### 2.3. Choice Test

To evaluate feeding and egg-laying preferences, we used only female leafhoppers—randomly selected from the stock population—which were offered a 24 h choice between paired systemic third leaves from differently treated plants. The following treatment combinations were established: C–OP, C–AVI, C–C and AVI–OP (Figure 1). Twenty-six repetitions were initiated per treatment combination; *n* = 99 trials were included in the statistical analysis. The exposed leaf sections were stained with acid fuchsin following Gerstenbrand et al. [33], after which salivary sheaths and eggs were counted under a stereomicroscope. Details of the choice-arena arrangement, leaf-section handling and staining/clearing procedure are provided in Supplementary Materials S3.

### 2.4. Feeding Behaviour Tests

Feeding behaviour tests were conducted using electropenetrography (EPG). We employed DC electropenetrographs (Giga-4 and Giga-2 models; Epgsystems Ltd., Wageningen, The Netherlands) to characterise the feeding behaviour of *P. alienus* on the differently treated plants. EPG was used to record and distinguish the durations of consecutive feeding phases, which were identified according to the waveform definitions provided by Tholt et al. [30] (Table 1). Consistent with the choice assays, treatments were applied to the second leaves of barley plants within micro-isolators 24 h before recording began. Recordings were performed on the systemic third leaves to evaluate plant-mediated effects. In each recording, EPG channels were randomly assigned to treatments.

To ensure a uniform level of starvation, leafhoppers were deprived of food for 1 h and tethered to the EPG electrode following Tholt et al. [30]; details of anaesthesia, wire attachment and electrode connection are provided in Supplementary Materials S5. During measurements, the insects were placed on the third systemic leaves for a 6 h recording period. For statistical analysis, the trial duration was defined as either 6 h—if the leafhopper was engaged in penetration at the designated end time—or as the time until the conclusion of the final active penetration.

Feeding behaviour was initially evaluated on 58 plants in each of the AVI and C treatments and on 116 plants in the OP treatment. The OP treatment was replicated more extensively because we expected greater variation in the amount of *Oulema* window-pane feeding than in the two standardised treatments. Final sample sizes were lower than these initial numbers because unsuccessful recordings—for example, when individuals died or were physically compromised during the assay—were excluded. Each EPG trial represented one independent biological replicate. We analysed feeding behaviour in two complementary ways. First, the damage type test compared the three treatment categories: C, AVI and OP. Second, within the OP treatment only, the damage intensity test assessed whether feeding behaviour varied with the quantified extent of actual *Oulema* window-pane feeding.

### 2.5. Statistical Analysis

All tests were performed in R 4.5.0 [34]. We used three distinct datasets to investigate how prior leaf damage inflicted by *Oulema* affected leafhoppers’ feeding and oviposition behaviour. The number of trials varied across the datasets. Model-specific sample sizes, inclusion criteria and treatment of right-censored observations are detailed in Supplementary Materials S6.

In the ‘Damage type test’ (*n* = 176 trials), we examined the feeding activity of leafhoppers assigned to the control group (C), artificial vascular injury group (AVI) or the *Oulema* pre-colonisation group (OP). Within the OP group, only trials where the beetle caused detectable leaf damage were included in the final dataset. Using this dataset, we investigated the effects of trial duration (as a standardised covariate), sex (as a factor, when both sexes were included), treatment (as a factor), and the interaction between sex and treatment on several response variables. We did not test treatment effects directly on waveform data; rather, by combining waveform information, we defined penetration-event variables that were biologically meaningful and suited to answer our research questions. We used the variables listed and explained in Table 1. Model-specific inclusion criteria, including the restriction of the phloem saliva ratio analysis to females, are provided in the Supplementary Materials S6. 

In models 4 and 6 (see Table 2 for model numbers), we applied mixed-effects Cox proportional hazards models using the ‘coxme’ R package [35]. In model 1, we fitted a generalised linear mixed model (GLMM) with negative binomial error distribution (and linear parameterisation) using Template Model Builder [36], whereas in all other cases, we fitted GLMMs with Gaussian error distributions. In models 3 and 5, we used arcsine transformation, and in models 7 and 8, we applied logarithmic transformation to improve the fit of the response variables to a normal distribution. In all models, we included ‘trial ID’ as the random term.

The dataset of the ‘Damage intensity test’ (*n* = 112 trials) comprised trials from the OP group, including trials in which *Oulema* did not damage the barley leaf during pre-colonisation. The distribution of *Oulema* damage intensity (% damaged area of the treated leaf section) was summarised in R using base functions for descriptive statistics, while skewness was quantified and tested with D’Agostino’s skewness test from the moments package, and departure from normality was assessed with the Shapiro–Wilk test [37]. In the damage intensity test, we examined the number of transitions (model 9); the percentage durations of non-penetration, non-phloem activities and phloem activity (models 10, 11, and 13, respectively); and the latencies to the first penetration and the first phloem activity (models 12 and 14, respectively). In models 12 and 14, we fitted mixed-effects Cox proportional hazards models. In model 9, a GLMM was applied using a negative binomial error distribution with linear parameterisation, whereas in all other cases, we fitted GLMMs with Gaussian error distributions. In model 10, the response variable was square-root-transformed. We included ‘trial ID’ as the random term in these models.

In analysing the ‘Choice test’ trials (*n* = 99 trials), we examined the difference in the numbers of salivary sheaths and eggs between the two available leaves in the treatment combinations (included as a factor) using GLMMs with Gaussian error distributions. In the latter case, the model included a zero-inflation formula with the fixed-effect predictor because of the high number of trials in which females laid no eggs. In both models, we included ‘trial ID’ nested in ‘date ID’ as the random term.

The proportional hazards assumption was tested using the ‘cox.zph’ function of the ‘survival’ R package [38,39] for all fitted Cox models. To check whether the assumptions of the GLMMs were met, we applied residual diagnostics using the ‘DHARMa’ R package [40]. To estimate the significance of potential predictors in the fitted mixed-effects models, we applied type III Wald χ^2^ tests using the Anova function of the ‘car’ R package [41]. Post hoc comparisons were performed with FDR (for comparisons to the theoretical null hypothesis) or Dunnett’s (for comparisons to the control group) adjustment using the ‘emmeans’ R package [42]. We used the relevant functions from this package to calculate estimated marginal means (EMMs) with corresponding 95% CIs and the ‘emtrends’ function to characterise the relationship between the total number of transitions and damage intensity separately in the two sexes. All tests were two-tailed, with α set to 0.05.

## 3. Results

### 3.1. Damage Type Test

**Figure 2:**
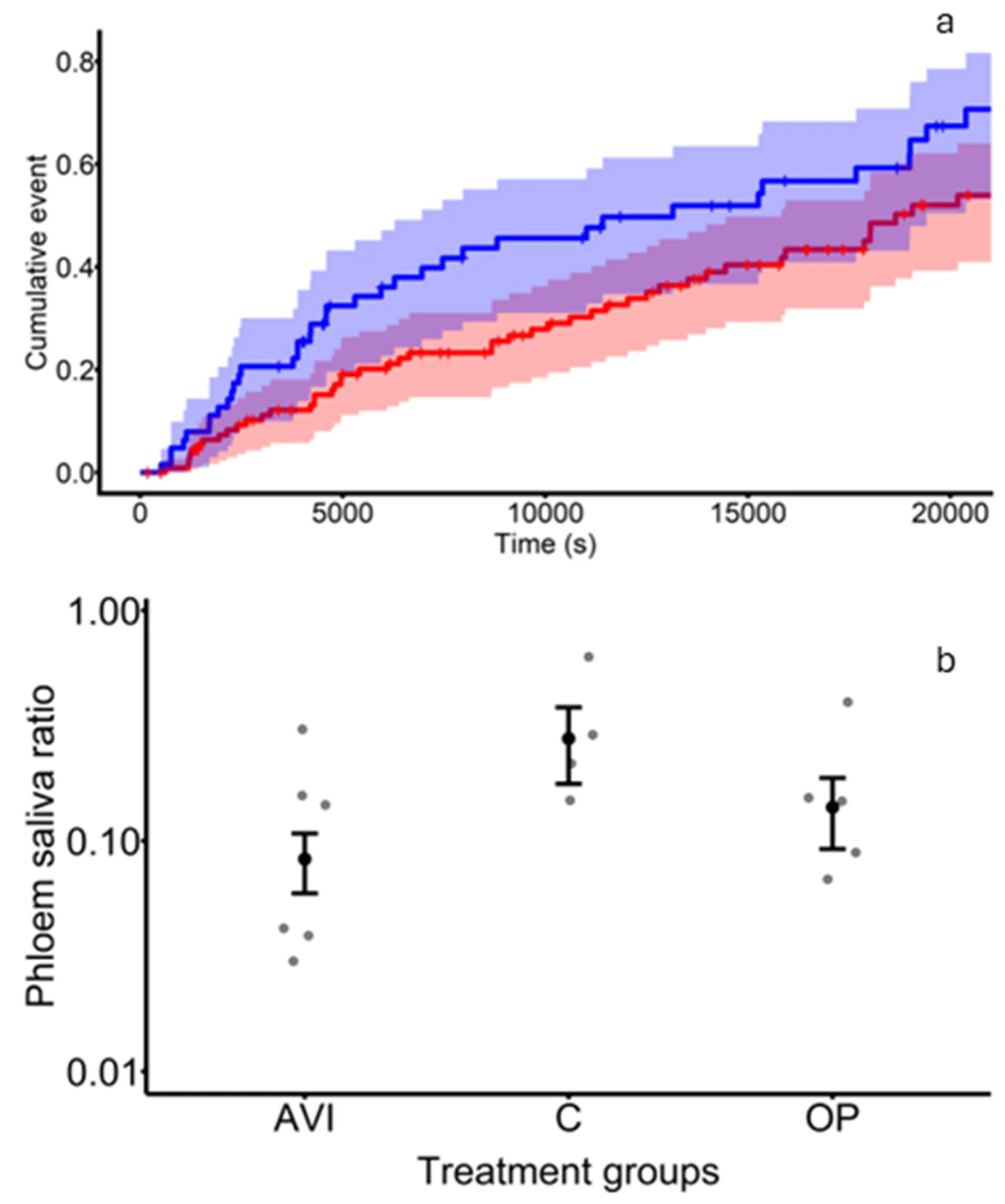
The effects of sex and treatment on leafhoppers’ feeding behaviour in the ‘Damage type test’. (**a**) Kaplan–Meier curves for the cumulative incidences of the phloem-penetration latencies in the two sexes (red: females; blue: males). Curves are shown with 95% confidence intervals. For graphical presentation only, we used the ‘survfit’ function of the ‘survival’ R package [38,39], without including the random term. (**b**) Differences between treatment groups in the phloem saliva ratio in females (on the log scale). Circles are jittered data points; estimated marginal means and corresponding standard errors were obtained from the fitted GLMM.

The treatment significantly affected phloem saliva ratio in females (*χ*^2^_2_ = 6.98, *p* = 0.031; Table 2; Figure 2a): this ratio was lower in the AVI group than in the C group (AVI: 0.08 [0.05; 0.15], C: 0.28 [0.14; 0.57], AVI/C contrast ratio ± SE = 0.3 ± 0.14, *t*-ratio = −2.63, *p* = 0.016) but did not differ between the C and OP groups (C: 0.28 [0.14; 0.57], OP: 0.14 [0.07; 0.27], 0.5 ± 0.26, *t*-ratio = −1.32, *p* = 0.321). We also found a significant difference between the sexes in phloem activity latency (*χ*^2^_1_ = 4.78, *p* = 0.029; Figure 2b), which indicates that males reached the phloem during feeding sooner than females (females: 0.83 [0.7; 0.98], males: 1.4 [1.04; 1.89], female/male contrast ratio ± SE = 0.59 ± 0.14, *z*-ratio = −2.19, *p* = 0.029; Figure 2b). The number of transitions between penetration phases, as well as the total time spent drinking, increased significantly with the length of the trial (both *χ*^2^_2_ ≥ 15.0, *p* < 0.001), but neither sex nor treatment nor the interaction of the two had any effect on these measures (all *p* ≥ 0.092). Moreover, we found no significant effect of any of the tested predictors on other response variables (all *p* ≥ 0.208, Table 2).

### 3.2. Damage Intensity Test

**Figure 3:**
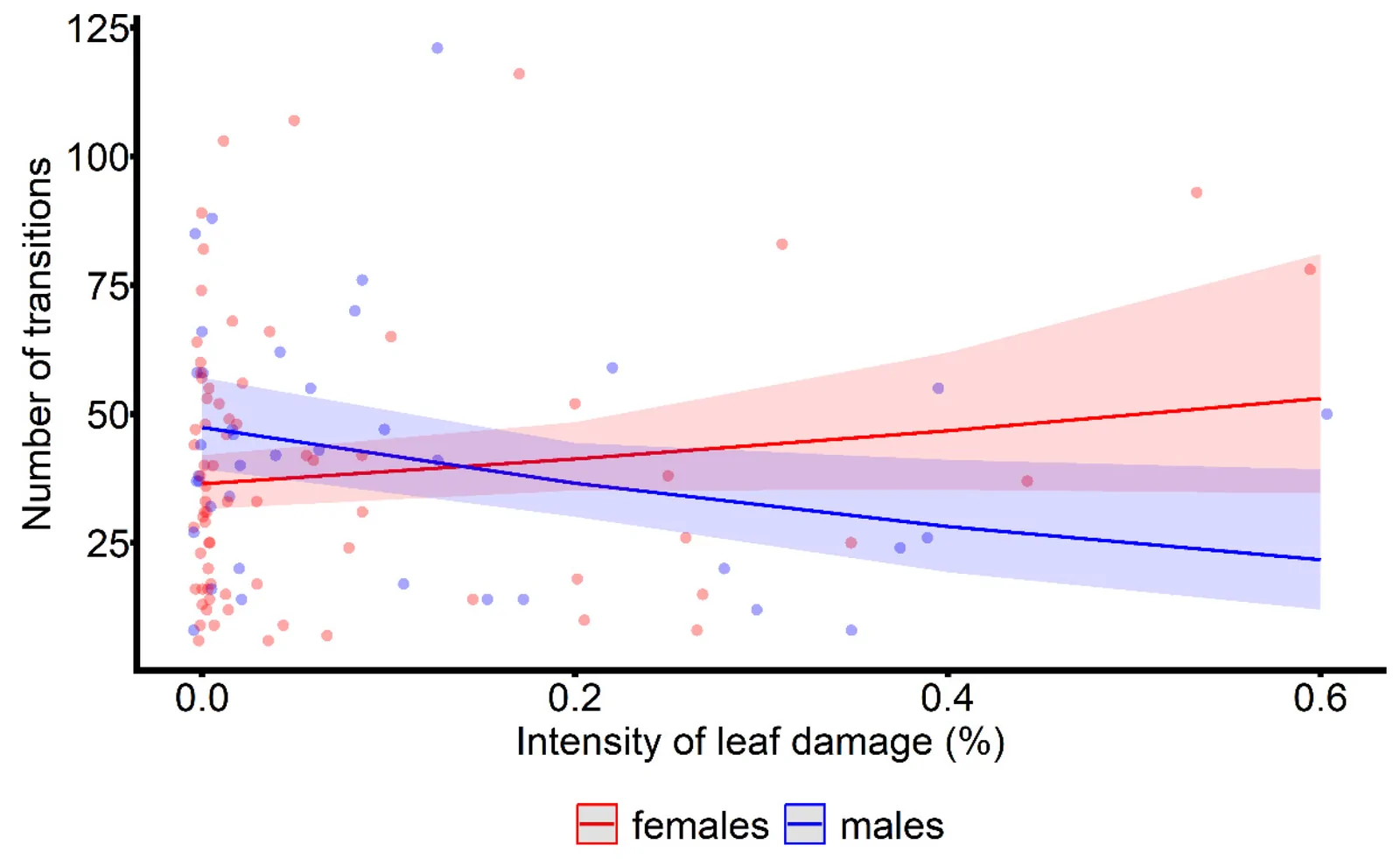
The relationship between the number of transitions and the intensity of *Oulema*-induced leaf damage in the two sexes (red: females; blue: males) in the ‘Damage intensity test’. Circles indicate jittered data points; regression lines denote predicted values (with bias correction) with 95% confidence intervals. The plot was generated using the ‘ggeffects’ R package [43].

*Oulema* damage intensity was generally low and strongly right-skewed (*n* = 112; mean ± SD = 8.30 ± 13.61%; median = 1.60%; range = 0.00–60.71%). Damage did not exceed 10% in 84 of 112 cases (75.0%). The skewness coefficient was 2.01, indicating significant positive skewness (D’Agostino z = 6.35, *p* < 0.001), and the distribution significantly departed from normality (Shapiro–Wilk W = 0.671, *p* < 0.001). The interaction of sex and damage intensity had a significant effect on the number of transitions between penetration phases in this experiment (*χ*^2^_1_ = 7.47, *p* = 0.006; Figure 3): damage intensity had a negligible influence on this measure in females but significantly decreased the total number of transitions in males (EMM of slope in females: 0.63 [−0.16; 1.41], males: −1.3 [−2.42; −0.17], female–male contrast = 1.92 ± 0.7, *z*-ratio = 2.73, *p* = 0.006). In the case of the other response variables, however, we found no significant effect of any of the tested predictors (all *p* ≥ 0.166, Table 3).

### 3.3. Female Choice Experiment

**Figure 4:**
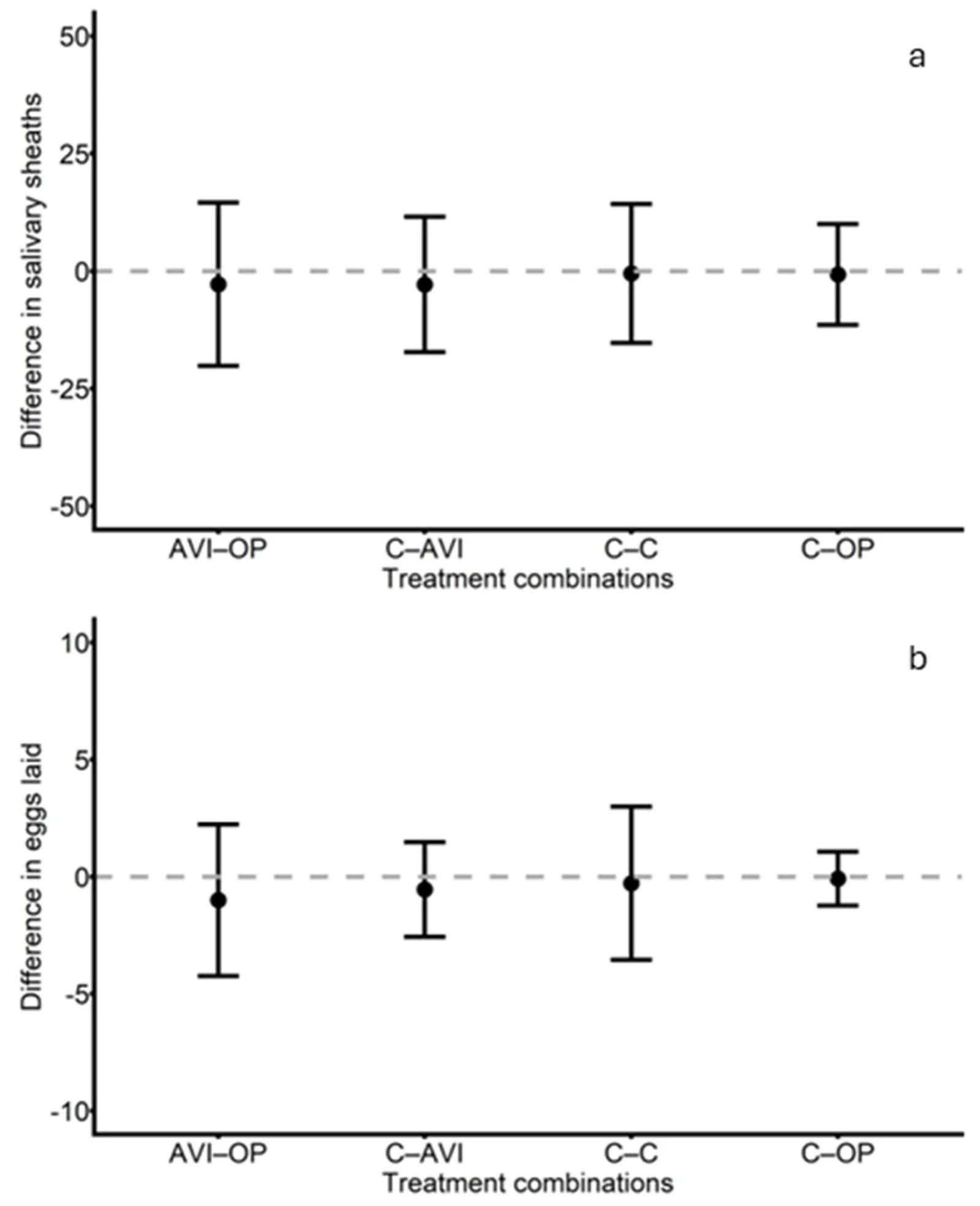
The effect of treatment combinations on female leafhoppers’ feeding and egg-laying behaviour in the ‘Female choice test’. (**a**) Difference in the number of salivary sheaths that females produced during feeding in each treatment combination. (**b**) Difference in the number of eggs laid on the available leaves in each treatment combination. In both panels, means with corresponding standard errors are shown; vertical dashed lines (in grey) indicate zero difference in the measured response between the two leaves with given treatments.

The difference in the number of salivary sheaths produced by female leafhoppers between the two leaves was similar in all treatment combinations (*χ*^2^_3_ = 0.8, *p* = 0.849; Figure 4a) and did not deviate from the theoretical null hypothesis (i.e., no difference between leaves) in the tested treatment combinations (all *p* ≥ 0.654). Similarly, we found no significant effect of these treatment combinations on the difference in the number of eggs laid on the two leaves (zero-inflation component: *χ*^2^_3_ = 1.58, *p* = 0.664; conditional component: *χ*^2^_3_ = 1.96, *p* = 0.582; Figure 4b). Deviations within each treatment combination were also consistent with the null hypothesis of no difference between the tested leaves (all *p* ≥ 0.173).

## 4. Discussion

Prior feeding by *Oulema* spp. produced only limited systemic effects on subsequent host use by *Psammotettix alienus* under our experimental conditions. In the EPG assays, beetle window-pane feeding did not alter most variables, including non-penetration, non-phloem activities, phloem activity, first penetration latency and phloem activity latency. The clearest treatment-level response was detected after artificial vascular injury: females feeding on artificially injured plants showed a lower phloem saliva ratio than females on control plants, whereas the *Oulema* pre-colonisation treatment yielded intermediate results that did not differ significantly from the control. In the damage intensity analysis, increasing *Oulema* damage reduced the number of transitions between penetration phases in males but not in females, while other feeding variables remained unaffected. The choice assay also provided no evidence that females discriminated among treatments in either salivary sheath production or oviposition. Taken together, these results indicate that the arrival sequence examined here—prior specialist window-pane feeding followed by a phloem-feeding vector on an undamaged systemic leaf—generated a weak plant-mediated legacy, closer to neutrality than to strong facilitation or competition.

This weak response is biologically informative because plant-mediated interactions among herbivores are often sequence-dependent, asymmetric and difficult to predict. Their magnitude and direction depend on herbivore identity, feeding mode, host plant and induction timing [4,5]. Previous studies have shown that earlier feeding can alter plant physiology and thereby modify the nutritional or defensive environment encountered by later colonisers. Comparable inhibitory effects have been reported across feeding guilds: prior or simultaneous chewing damage reduces aphid performance in potato [6], milkweed [5], and safflower [44] systems. However, such effects are not universal; in maize, for example, caterpillar-induced aphid resistance varies among genotypes and plant defence chemistry [45]. In the present study, plant physiological and defence responses were not measured directly, so the mechanisms underlying the weak behavioural response in our study system remain unresolved. Nevertheless, the results indicate that prior *Oulema* window-pane feeding did not markedly impair the later phloem-feeding vector when assessed systemically after a 24 h induction period.

The distinction between artificial vascular injury and authentic *Oulema* feeding is central to interpreting these results. The artificial injury treatment was not designed as a mechanical surrogate for beetle herbivory; instead, it imposed a standardised vein-breaching injury that severed smaller parallel veins on one side of the monocot leaf. By contrast, *Oulema* window-pane feeding removes epidermal and mesophyll tissue between the veins while largely sparing the vascular elements that *P. alienus* ultimately needs to reach. This difference may explain why artificial vascular injury, but not *Oulema* feeding, altered the phloem saliva ratio. Accordingly, even limited vascular injury may produce a detectable systemic effect on phloem-feeding behaviour, whereas interveinal window-pane feeding, potentially influenced by herbivore-associated cues, appears to leave a weaker systemic legacy under our conditions. Our artificial injury treatment should, therefore, be viewed as an informative contrast between mechanical vascular injury and specialist window-pane feeding with herbivore-derived cues, rather than as a surrogate for *Oulema* herbivory.

Population-level evidence is also consistent with weak or context-dependent interactions between cereal leaf beetles and sap feeders. In wheat fields, aphid and cereal leaf beetle abundances were explained mainly by wheat phenology and landscape variables rather than direct aphid–*Oulema* effects [46]. Wetzel et al. [47] found intraspecific competition within each pest group but no adverse interspecific density effect between cereal aphids and *O. melanopus*; combined infestation caused a less-than-additive yield loss, attributed to plant compensation rather than antagonism between the pests. Damage in our experiment was at the low end of field-relevant CLB injury: treated leaf sections showed a strongly right-skewed distribution, with a mean of 8.30% and a median of 1.60%. On 75% of the experimental plants, leaf damage did not exceed 10% of the exposed area. This falls within the range of moderate field injury, such as the 14% mean leaf damage reported in Hungarian wheat fields [48]. Field damage can, nevertheless, be highly uneven: Samu et al. [17] found a median of zero larvae per plant but >35 larvae on 10% of plants, with patchiness explaining 40% of CLB damage variance, while outbreak damage may reach 85–90% leaf area loss [49,50]. Against this background, our weak systemic response probably characterises low-to-moderate damage due to short-term feeding, whereas severely damaged patches or outbreak years may produce stronger plant-mediated effects.

The phloem saliva ratio was the clearest treatment-level EPG response, reflecting the proportion of phloem activity devoted to salivation rather than ingestion and, thus, the relative investment required for sustained sap uptake [51]. The pattern of C > OP > AVI indicated reduced phloem salivation in females on artificially injured plants, whereas the *Oulema* treatment was intermediate and did not differ from the control. Because phloem activity latency and total phloem activity were unchanged, this represented a narrow shift in phloem-phase allocation rather than faster access or greater phloem use, consistent with Wang et al. [14], where prior chewing damage altered sap-feeder phloem-phase investment rather than initial phloem access latency. Similarly, increasing *Oulema* damage reduced transitions between penetration phases in males, which suggests more stable probing, but this sex-specific response was not accompanied by changes in non-penetration, non-phloem activities, phloem activity or phloem activity latency [52]. The absence of treatment effects on non-phloem activities and phloem activity is, therefore, important because it suggests that, under the different treatment conditions, the leafhoppers maintained the core behavioural components of salivary sheath feeding [3,25,30] and nutrient acquisition from sugar-rich but nitrogen-limited phloem sap [10].

The relatively weak behavioural response to *Oulema* pre-colonisation helps place the present results within the response gradient suggested by previous EPG studies on *P. alienus*. On non-host plants, probing was strongly disrupted: on a dicot, *P. alienus* failed to reach the phloem, whereas on a monocot, it reached the phloem but did not achieve sustained ingestion [30]. Under predator risk, the response was less pronounced, with reduced penetration activity, shorter phloem preparation, and delayed or less frequent phloem ingestion, while drinking was delayed but not reduced [25]. The present study extends the weaker end of this gradient: artificial vascular injury caused a narrow reduction in relative phloem salivation, whereas *Oulema* feeding produced only a sex-specific change in phase transitions. Thus, *P. alienus* appears to respond most strongly to unsuitable host identity, intermediately to non-consumptive predator risk, and weakly to short-term systemic effects induced by prior herbivory. This pattern may reflect the need to preserve essential physiological functions, particularly water balance and osmoregulation under the high sugar load of phloem sap [53]. This interpretation is consistent with previous findings that even feeding on non-host plants can increase survival relative to complete starvation [30].

The choice experiment likewise indicated that prior *Oulema* damage did not cause a strong systemic change in host acceptability: female leafhoppers showed no preference among treatments in salivary sheath production or oviposition. Although *Oulema* feeding may induce plant cues [22], any such systemic effect after 24 h was insufficient to alter leafhopper behaviour. Thus, whatever changes may have been induced by prior feeding, they were not strong enough under our experimental conditions to override the basic physiological requirements of *P. alienus*. This result does not exclude stronger effects after longer induction periods or on locally damaged leaves. In particular, severe window-pane feeding could directly reduce the intact leaf surface available for settlement or egg insertion [17], a local effect not addressed by our systemic assay.

The epidemiological implications should be interpreted in a measured and feeding-phase-specific context. WDV inoculation is associated with phloem salivation, whereas virus acquisition occurs during phloem ingestion [25]. Because these phases represent the feeding-related opportunities for the two transmission processes, changes in their occurrence, duration or relative allocation are necessarily relevant to transmission potential. In the present study, artificial vascular injury altered the relative allocation between phloem salivation and ingestion, whereas the response to *Oulema* pre-colonisation was intermediate and did not differ significantly from the control. These EPG responses should, therefore, be interpreted as transmission-relevant behavioural changes rather than direct evidence of altered virus transmission. Their effects on realised acquisition, inoculation efficiency and field-level WDV spread cannot be quantified from the present data. Nevertheless, experimental and modelling studies indicate that even modest changes in the vector’s feeding or host-use behaviour can scale up to altered pathogen spread, making these behavioural effects relevant targets for future epidemiological investigation [54,55].

## 5. Conclusions

Cereal crops are transient and dynamic habitats in which herbivore arrival sequence can shape plant-mediated interactions, but our results show that such effects are not necessarily strong, negative or easily predicted from feeding guild alone. Prior interguild damage by *Oulema* did not impair P. alienus feeding behaviour under the short-term systemic conditions tested here; instead, window-pane feeding produced a largely neutral behavioural outcome, with only limited signs of altered feeding organisation of the leafhopper. By contrast, artificial vascular injury caused a more detectable but still narrow shift in phloem-phase allocation, which suggests that vein-breaching damage may reduce the relative salivary investment required once the phloem has been reached. This contrast highlights the importance of damage type: specialist interveinal window-pane feeding, vein-damaging chewing injury and other forms of “chewing herbivory” may differ substantially in their consequences for vascular integrity, defence induction and later phloem-feeder behaviour. The high number of EPG and choice-test replicates strengthens confidence that a strong treatment effect was not missed; thus, the absence of broad behavioural responses should be treated as informative rather than inconclusive. Nevertheless, because *Oulema* damage intensity was at the lower end of field-observed values and responses were measured after a 24 h systemic induction period, stronger damage, longer induction, repeated herbivory or local feeding sites may produce different outcomes. More broadly, the characteristic window-pane feeding mode of *Oulema*, which removes epidermal and mesophyll tissues while largely sparing vascular tissues, may allow partial spatial separation from phloem-feeding insects, consistent with population-level evidence for limited apparent competition between cereal leaf beetles and cereal sap feeders. Future work should combine EPG with local versus systemic assays, plant-chemistry measurements and direct WDV acquisition or inoculation experiments to determine whether the subtle behavioural shifts detected here scale up to altered virus-transmission risk in cereal agroecosystems.

## Figures and Tables

**Table 1:** Summary of EPG waveforms and derived variables used to analyse the feeding behaviour of *Psammotettix alienus*.

**Waveform**	**Phase name**	**Biological meaning**
NP	Non-penetration	All phases without penetration
Ps1	Pathway	Salivary sheath secretion and search
Ps3	Pathway	Watery saliva secretion in mesophyll
Ps2	Xylem ingestion	Drinking
Ps4a	Phloem saliva secretion (X wave)	Phloem saliva secretion (X wave)
Ps4b	Phloem ingestion (feeding)	Phloem ingestion (feeding)
**Waveform combination**	**Variable name and definition**	**Biological meaning**
any	*Transitions*: total number of transitions between phases	Represents the stability (few transitions) versus instability (many transitions) of the feeding
Ps1+Ps2+Ps3	*Non-phloem activities*: percentage duration of non-phloem activities	Represents phloem searching and drinking, i.e., activities occurring outside the phloem
NP	*Non-penetration*: Percentage duration of non-penetration	Reflects the amount of time spent without penetration
Ps1	*First penetration* *latency*	Indicates the latency to the first stylet penetration
Ps4a+Ps4b	*Phloem activity*: percentage duration of phloem penetration	Indicates the total time spent with preparation (watery saliva) and ingestion in the phloem
Ps4a	Phloem activity latency: latency to first Ps4a	Indicates the time to first reach the phloem
Ps4a/Ps3	*Saliva secretion location*: ratio represents the duration of saliva secretion in the phloem relative to that in the mesophyll	May indicate whether responses to induced plant changes are expressed primarily in the mesophyll or phloem
Ps4a/(Ps4a+Ps4b)	*Phloem saliva ratio*: the ratio of phloem saliva secretion to total phloem activities	Indicates the relative ‘investment’ in phloem salivation within total phloem activity

**Table 2:** Test statistics and the significance of the explanatory variables from the mixed-effect models fitted on the investigated behavioural parameters in *Psammotettix alienus* in the ‘Damage type test’. Significant predictors in the final models and their values are shown in bold. Test statistics and *p*-values for the non-significant predictors were computed by including them one by one in the final models.

Model	Response Variable	Predictors	*χ* ^2^	df	*p*
1	Transitions	**Length of trial**	**83.42**	**1**	**<0.001**
		Sex	0.03	1	0.868
		Treatment	3.73	2	0.155
		Sex × Treatment	4.78	2	0.092
2	Non-penetration	Length of trial	1.58	1	0.208
		Sex	0.26	1	0.608
		Treatment	2.32	2	0.314
		Sex × Treatment	0.37	2	0.832
3	Non-phloem activities	**Length of trial**	**15.0**	**1**	**<0.001**
		Sex	0.1	1	0.758
		Treatment	0.06	2	0.968
		Sex × Treatment	1.4	2	0.497
4	First penetration latency	Length of trial	0.62	1	0.431
		Sex	0.04	1	0.844
		Treatment	1.38	2	0.502
		Sex × Treatment	0.33	2	0.849
5	Phloem activity	Length of trial	1.87	1	0.171
		Sex ^†^	0.09	1	0.759
		Treatment	0.94	2	0.625
		Sex × Treatment	0.33	2	0.848
6	Phloem activity latency	Length of trial	0.06	1	0.799
		**Sex**	**4.78**	**1**	**0.029**
		Treatment	3.97	2	0.137
		Sex × Treatment	1.55	2	0.461
7	Saliva secretion location (Ps4a/Ps3)	Length of trial	0.06	1	0.802
		Sex	0.41	1	0.522
		Treatment	1.67	2	0.434
		Sex × Treatment	1.36	2	0.506
8	Phloem saliva ratio	Length of trial	<0.01	1	0.959
		**Treatment**	**6.98**	**2**	**0.031**

^†^ GLMM fitted poorly to the data with this variable as the only predictor (Kolmogorov–Smirnov test for normality: *p* = 0.06).

**Table 3:** Test statistics and the significance of the explanatory variables from the mixed-effect models fitted on the investigated behavioural parameters in *Psammotettix alienus* in the ‘Damage intensity experiment’. Significant predictors in the final models and their values are shown in bold. Test statistics and *p*-values for the non-significant predictors were computed by including them one by one in the final models.

Model	Response Variable	Predictors	*χ* ^2^	df	*p*
9	Transitions	**Length of trial**	**75.48**	**1**	**<0.001**
		**Sex**	**5.67**	**1**	**0.017**
		Damage	2.46	1	0.117
		**Sex × Damage**	**7.47**	**1**	**0.006**
10	Non-penetration	Length of trial	0.01	1	0.94
		Sex	0.44	1	0.508
		Damage	0.06	1	0.813
		Sex × Damage	0.02	1	0.878
11	Non-phloem activities	Length of trial ^††^			
		Sex ^††^			
		Damage	<0.01	1	0.975
		Sex × Damage	0.78	1	0.377
12	First penetration latency	Length of trial	0.02	1	0.888
		Sex	0.44	1	0.51
		Damage	0.16	1	0.691
		Sex × Damage	0.83	1	0.363
13	Phloem activity	Length of trial ^††^			
		Sex	0.54	1	0.463
		Damage	0.02	1	0.893
		Sex × Damage	0.1	1	0.757
14	Phloem activity latency	Length of trial	0.08	1	0.773
		Sex	1.92	1	0.166
		Damage	1.51	1	0.219
		Sex × Damage	0.03	1	0.868

^††^ GLMM fitted the data badly when only this predictor was present, so its effect was not estimated.

## Data Availability

All research data are available via Figshare (https://doi.org/10.6084/m9.figshare.33094592).

## Supplementary Materials

The following supporting information can be downloaded at: https://www.mdpi.com/article/10.3390/insects17080783/s1. Supplementary Materials S1. Plant and insect maintenance; Supplementary Materials S2. Construction and use of the micro-isolators; Supplementary Materials S3. Detailed arrangement of the choice assay; Supplementary Materials S4. Quantification of *Oulema*-induced leaf damage; Supplementary Materials S5. Preparation of leafhoppers for EPG recording; Supplementary Materials S6. Sample sizes and model-specific inclusion criteria; Table S1: Descriptive statistics of some trial characteristics and the durations of different waveforms.
