# Supplementary material for: Prior Cereal Leaf Beetle (Oulema spp.) Window-Pane Feeding Has Weak Systemic Legacy on the Wheat Dwarf Virus Vector Psammotettix alienus in Barley
Source: Insects. 2026 Jul 28;17(8):783. doi: 10.3390/insects17080783 (PMC13513543; doi:10.3390/insects17080783)
Supplement: Supplementary file 1 [file insects-17-00783-s001.zip › insects-4414090-supplementary.pdf]

Supplementary Materials

Supplementary Materials S1. Plant and insect maintenance

To maintain the stock population of *Psammotettix alienus*, approximately ten seeds of barley (*Hordeum vulgare* cv. Mv. Conchita) were sown in 1.2 L pots. Leafhoppers were introduced once the plants had reached the tillering stage. The pots were covered individually with mesh cages with a mesh size of 0.2 mm and maintained in a climate chamber at 21 °C under an L16:D8 photoperiod.

Adult cereal leaf beetles were collected from a 1.5 × 1.5 m experimental barley plot managed specifically to attract naturally occurring *Oulema* spp. from the surrounding landscape. After collection, the beetles were maintained on mesh-covered barley plants in a climate chamber under the same temperature and photoperiod conditions as the leafhopper stock population. This acclimation ensured that the experimental beetles were exposed to the same temperature and light regime as the host plants before the assays.

Supplementary Materials S2. Construction and use of the micro-isolators

Custom micro-isolators were constructed from sections of plastic binding comb, each approximately 33 mm in diameter and 50 mm in length, and lined with fine mesh to prevent insect escape. The micro-isolators enclosed a defined section of the second leaf and thereby standardised the leaf area exposed to the treatments.

A micro-isolator was attached to the second leaf of every experimental plant, including plants assigned to the undisturbed control treatment. The isolators remained in place for the entire 24 h treatment period.

Supplementary Materials S3. Detailed arrangement of the choice assay

For each choice assay, two treated plants were positioned side by side in their original pots. Their untreated systemic third leaves were placed parallel to one another on an experimental platform, with the adaxial surfaces facing upwards. A single female *P. alienus* was released onto the paired leaves and confined within a circular arena formed by a Petri dish lid. The lid delimited the accessible feeding area and prevented the insect from escaping during the 24 h assay.

At the end of each trial, the leafhopper was removed and the leaf sections exposed during the assay were excised. The sections were placed in phosphate-buffered saline in Eppendorf tubes and stored at 5 °C until processing. The leaves were subsequently stained with 5% acid fuchsin to visualise salivary sheaths and eggs, cleared in 80% lactic acid and stored in glycerine. Salivary sheaths and eggs were counted under a stereomicroscope.

Supplementary Materials S4. Quantification of *Oulema*-induced leaf damage

Following completion of the choice and EPG assays, the treated second leaves from plants assigned to the *Oulema* pre-colonisation treatment were photographed for damage quantification. The leaf sections were back-illuminated to increase the contrast between the consumed parenchyma and the remaining intact epidermis.

Digital images were analysed using ImageJ software. The area showing window-pane feeding damage was delineated and expressed as a percentage of the total leaf area enclosed within the micro-isolator. This percentage was used as the damage-intensity variable in the subsequent analyses.

Supplementary Materials S5. Preparation of leafhoppers for EPG recording

Leafhoppers were removed from the stock plants exactly 1 h before the beginning of the EPG recordings to standardise the period without access to food. During this period, the insects were anaesthetised with CO_2 and tethered to a fine gold wire of approximately 20 µm diameter using conductive silver glue,

following the procedure described by Tholt et al. [30]. The gold wire was subsequently connected to the insect electrode of the electropenetrograph.

During recording, each tethered leafhopper was positioned on the untreated systemic third leaf of the experimental plant. Each insect was used in only one recording.

Supplementary Materials S6. Sample sizes and model-specific inclusion criteria

Each EPG trial represented one independent biological replicate, consisting of one leafhopper tested once on one plant. Although the waveform-level datasets contained multiple rows per individual, these rows represented repeated feeding events within the same trial and were not treated as independent replicates.

The damage-type analysis included 176 trials from the control (C), artificial vascular injury (AVI), and *Oulema* pre-colonisation (OP) treatments. OP trials were included only when detectable *Oulema* damage had occurred, because the purpose of this analysis was to compare plants exposed to actual beetle damage with the other treatments. All 176 trials were used in models 1–4. Model 5 included the 83 trials in which phloem-related activity occurred, because phloem activity could only be evaluated for leafhoppers that reached this feeding phase. In model 6, all 176 trials contributed to the latency analysis; 83 contained an observed first phloem-salivation event, while trials in which this event did not occur before the end of the recording were retained as right-censored observations rather than excluded.

The ratio variables required the occurrence of specific waveform phases. Model 7 included 59 trials in which the waveform durations required to calculate the Ps4a/Ps3 ratio were available. Model 8 included 15 trials in which both phloem salivation and a completed phloem-ingestion phase were recorded, allowing calculation of Ps4a/(Ps4a + Ps4b). Trials lacking either required phase, or containing a censored duration that prevented calculation of the ratio, were excluded from the respective analysis. Because only two control males reached Ps4b, model 8 was restricted to females.

The damage-intensity analysis included 112 OP trials for which damaged leaf area was quantified. In contrast to the damage-type analysis, trials with zero detectable damage were retained because damage intensity was analysed as a continuous variable. All 112 trials were used in models 9–12. Model 13 included the 51 trials in which phloem-related activity occurred. In model 14, all 112 trials contributed to the latency analysis; 51 contained an observed first phloem-salivation event, while trials in which this event was not observed during the recording were treated as right-censored observations.

Supplemetary Results

Table S1. Descriptive statistics of some trial characteristics and the durations of different waveforms.

Characteristics/waveforms	*N*	Mean ± SD
Trial duration (s)	176	15798.78 ± 6921.03
Intensity of *Oulema* leaf damage (%)	112	8.3 ± 13.61
NP (%)	176	42.23 ± 22.82
Ps1 (%)	176	20.72 ± 15.06
Ps3 (%)	134	17.11 ± 15.63
Ps2 (%)	172	15.18 ± 15.60
Ps4a (%)	83	4.41 ± 4.32
Ps4b (%)	41	30.53 ± 19.85
